# Alpha-gal sensitization and allergic blood transfusion reactions: a scoping review

**DOI:** 10.1186/s12967-025-07614-9

**Published:** 2026-02-04

**Authors:** Maureen J. Miller, Roya Zarpak, Patricia Lee, Zachary Osterwind, Mattias Lenz, Mirna Argueta Guevara, Sarah Fowler, Carrie Price, Tracy C. Shields, Alicia A. Livinski, Kathleen Conry-Cantilena, David F. Stroncek, Kamille West-Mitchell, Valeria De Giorgi

**Affiliations:** 1 NIH Clinical Center, Department of Transfusion Medicine, 10 Center Drive, Bethesda, Maryland, USA; 2 NIH Library, Office of Research Services, Office of the Director, National Institutes of Health, Bethesda, MD USA

**Keywords:** alpha-gal syndrome, alpha-gal sensitization, blood donor testing, tickborne illnesses, transfusion-transmitted infections, transfusion-related alpha-gal syndrome (TRAGS)

## Abstract

**Background:**

Tick bites may expose individuals to a carbohydrate not found in humans, galactose-alpha-1,3-galactose (alpha-gal), including allergy to mammalian red meat or red meat-derived products presenting 2–6 hours after consuming the product plus positive alpha-gal specific IgE testing that may indicate alpha-gal syndrome (AGS). Anaphylaxis in group O recipients of group B blood products in the absence of other risk factors for allergic transfusion reactions could be transfusion-related alpha-gal syndrome (TRAGS); galactose-alpha-1,3-galactose (Gal-alpha1-3 Galβ1-(3)4GlcNAc-R) is similar to the B blood group antigen (Gal-alpha1-3(Fuc-alpha-1,2)Gal).

**Objectives:**

To review scientific and grey literature with no date or language limit to: 1) describe characteristics of all known cases of transfusion-related alpha-gal syndrome (TRAGS) and hypersensitivity reactions to infusions of mammalian red meat-derived medical products besides blood components that may resemble TRAGS; 2) identify studies that explore possible relationships between alpha-gal sensitization and blood group that may be relevant to understanding TRAGS; 3) describe which clinical, laboratory, and epidemiologic parameters used to diagnose AGS food allergy are also appropriate to diagnose TRAGS; and 4) identify which diagnostic assays exist for AGS and how they are used for AGS and/or TRAGS.

**Methods:**

Using search strategies peer-reviewed by biomedical librarians we performed a scoping review of literature in five medical databases, charted in Microsoft Excel and Covidence software.

**Results:**

Of 11732 studies imported for screening, 3858 duplicates were removed and 7874 papers were screened. Title and abstract screening excluded 7383 papers. Full-text screening excluded 380 papers. Of the included papers, 20/111 (18%) addressed risk for alpha-gal allergy by blood group and 18/111 diagnostics (16%).

**Conclusion:**

A comprehensive literature review showed a possible association between alpha-gal allergy and blood group. Further guidance is needed regarding potential clinical implications.

**Supplementary Information:**

The online version contains supplementary material available at 10.1186/s12967-025-07614-9.

## Introduction

Tick bites from species including *Ambylomma americanum* (lone star tick), *Ixodes* species, and *Haemaphysalis longicornis* (Asian long-horned tick) induce IgE antibodies to a carbohydrate not found in humans, galactose-alpha-1,3-galactose (alpha-gal) [1–13]. Alpha-gal syndrome (AGS), an allergy to mammalian red meat or red meat-derived products, may result from IgE-mediated hypersensitivity reactions to exposure to alpha-gal and could possibly affect thousands of people [14]. The clinical diagnosis of AGS, as opposed to alpha-gal sensitization alone, is based on a characteristic history of delayed allergic reactions (typically 2–6 hours) after mammalian red meat ingestion, detectable alpha-gal-specific IgE (≥0.1 kU/L), and resolution or significant improvement of symptoms with dietary avoidance of mammalian red meat or mammalian red meat-derived products. No single test is diagnostic without compatible clinical history [15]. There is a clinical spectrum of AGS reactions. AGS reactions vary widely in symptomatology (e.g., gastrointestinal symptoms, arthritis, pruritis) [16], severity [17, 18], and geographic region [7]. Importantly, individuals with alpha-gal sensitization may not have symptomatic AGS [19]. Risk factors for AGS include older age, male sex, Caucasian race, other allergies (e.g., milk, egg, fish, animals, cat dander) [10, 20–29], and recurrent tick bites [30–33] outdoors [34]. Patients with AGS show higher anti-alpha-gal IgE levels [23, 24, 35–38] which may wane [1, 39] or not correlate with symptom severity [11, 27].

AGS-like symptoms have been observed in people following transfusion with allogeneic (healthy donor) blood products. Recent reports of anaphylaxis in group O recipients of group B plasma in the absence of other risk factors for severe allergic transfusion reactions (ATRs) (e.g., IgA deficiency) could indicate alpha-gal sensitization [40], though these transfusion case reports lack dietary challenge or avoidance data. All the reported cases were severe and came from regions of the United States with a high prevalence of AGS compared to the spectrum of AGS reactions seen elsewhere. An association between blood group and alpha-gal sensitization seems biologically plausible: The allergen galactose-alpha-1,3-galactose (Gal-alpha1-3 Galβ1-(3)4GlcNAc-R) is antigenically similar to the B blood group antigen (Gal-alpha1-3(Fucalphal,2)Gal). Thus, potential cross-reactivity of alpha-gal specific IgE to B red blood cells may pose a safety consideration for selection of blood products for transfusion [40, 41]. The objective of this study was to perform a scoping review of publications on alpha-gal sensitization with no date or language limit to:

1. Describe characteristics of all known cases of transfusion-related alpha-gal syndrome (TRAGS) and hypersensitivity reactions to infusions of mammalian red meat-derived medical products besides blood components that may resemble TRAGS;
2. Identify studies that explore possible relationships between alpha-gal sensitization and blood group that may be relevant to understanding TRAGS;
3. Describe which clinical, laboratory, and epidemiologic parameters used to diagnose AGS food allergy are also appropriate to diagnose TRAGS;
4. Identify what diagnostic assays exist for AGS and how they are used for AGS and/or TRAGS.

## Methods

### Human subjects research and ethics

This study did not use human subjects. Institutional review board and ethics approval were not required.

### Protocol and review methodology

The scoping review followed the methodology described in the *JBI Manual for Evidence Synthesis* [42]. A study protocol was prepared in accordance with the JBI methodology [43]. PRISMA Extension for Scoping Reviews (PRISMA-ScR) was the reporting guideline [44] (Additional Files, Supplemental Material 1).

### Study objectives (research questions)

The research questions were as follows:

1. What are the characteristics of all known cases of transfusion-related alpha-gal syndrome (TRAGS) and hypersensitivity reactions to infusions of mammalian red meat-derived medical products besides blood components that may resemble TRAGS?
2. Do studies show a possible relationship between alpha-gal sensitization and blood group, and is it relevant to understanding TRAGS?
3. Which clinical, laboratory, and epidemiologic parameters used to diagnose AGS food allergy are also appropriate to diagnose TRAGS?;
4. What diagnostic assays exist for AGS and how are they used for AGS and/or TRAGS?

### Eligibility criteria

*Inclusion criteria.* This broad scoping review considered all studies that focus on AGS or alpha-gal sensitization AND blood. The population was all persons reported in the literature; the exposures of interest were any causing alpha-gal sensitization; the outcomes of interest were evidence of TRAGS (provisionally defined by Dunbar et al. in 2025 as a state “in which patients with AGS may be at risk for severe and even fatal ATRs following exposure to group B antigen and/or substance through plasma or platelet transfusions” [41]) or unnamed, unexplained allergic, anaphylactic, or anaphylactoid transfusion reactions in patients who received out-of-group blood transfusions after the clinical characterization of anaphylaxis from alpha-gal sensitization circa 2007 before TRAGS was named. We included published studies, scientific commentaries, and grey literature with no date limit. Studies published in non-English languages were reviewed with Google Translate.

*Exclusion criteria.* All citations that failed to meet the inclusion criteria were excluded.

### Information sources and search strategy

A biomedical librarian searched PubMed (NCBI), which includes MEDLINE; Embase (Embase.com); Cochrane Database of Systematic Reviews and Cochrane Central Register of Controlled Trials (Wiley); Scopus (Elsevier); and Web of Science Core Collection (Clarivate; SCI-EXPANDED [1900 – present], SSCI [1900 – present], CPCI-S [1990 – present], CPCI-SSH [1990 – present], BKCI-S [2005 – present], BKCI-SSH [2005 – present], ESCI [2005 – present], and CCR-EXPANDED [1985 – present], IC [1993 – present]) (Additional Files, Supplemental Material 2).

### Software

The citation management software used were EndNote 20 and 21 (Clarivate Analytics, Philadelphia, PA, USA and London, UK) and Covidence (Veritas Health Innovation, Melbourne, Australia). Data collection was performed in Covidence, statistical analyses in Covidence and Microsoft Excel (Microsoft Corporation, Redmond, Washington, USA), and data visualization in GraphPad Version 10.6.1 for Windows (GraphPad Software, LaJolla, California, USA). An image in this manuscript was designed in Biorender (Toronto, Ontario, Canada).

### Study selection and screening

We used a two-stage screening process in Covidence. First, at least two independent reviewers screened all titles and abstracts retrieved for eligibility criteria. If the reviewers found any ambiguous information or disagreed about an article’s inclusion or exclusion, an additional reviewer screened the article as a tiebreaker. Second, two independent reviewers screened the full texts associated with all included titles and abstracts. The first and senior authors made the final decision to include or exclude the citation in the results.

### Data collection and data items

*Data collection*. We used a data charting form developed by health information scientists to pilot data collection [45], then developed a form specific to our review in Covidence (Additional Files, Supplemental Material 3).

*Data Items.* We extracted these data: title; publisher; corresponding author; geographic area(s) where the study was conducted; study characteristics (methods, start and end date of data collection, study funding sources, possible conflicts of interest for study authors); study participants (recruitment; inclusion and exclusion criteria; total number of participants; population characteristics); major results; conclusion; study strengths and weaknesses; and the reviewer’s subjective impression of the quality of the publication:

1. Relevant (discusses alpha-gal sensitization and blood);
2. Potentially relevant (discusses alpha-gal sensitization and clinical outcomes possibly related to blood);
3. Missing information (discusses alpha-gal sensitization with no reference to blood);
4. Not relevant (no discussion of alpha-gal sensitization); or
5. Unknown (all other papers).

### Data analysis & synthesis

**Fig. 1:**
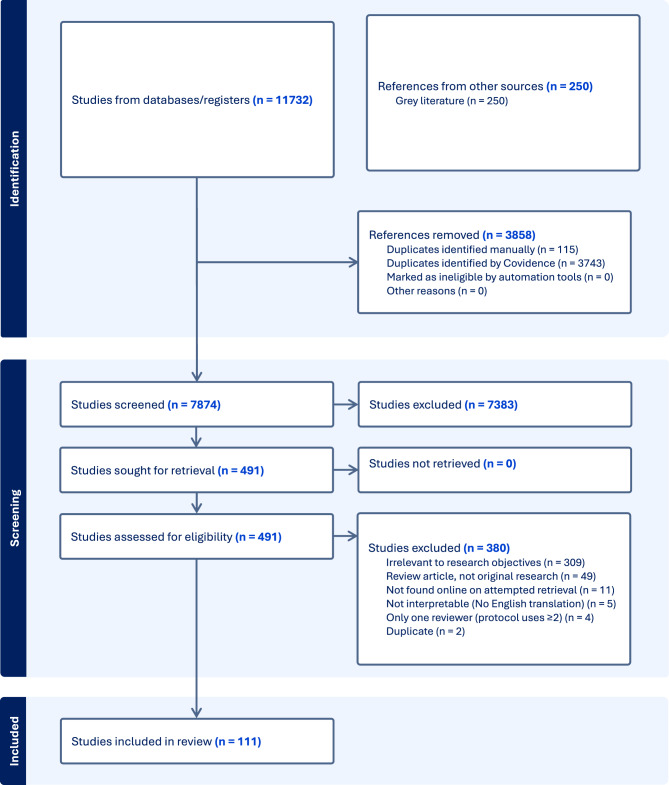
PRISMA flow diagram of inclusion and exclusion criteria (Covidence). This flow diagram summarizes inclusion and exclusion criteria for all citations used in a comprehensive scoping literature review according to Preferred Reporting Items for Systematic reviews and Meta-Analyses extension for scoping reviews (PRISMA-ScR) criteria. Reviewers screened the articles in a two-step process (title and abstract screening, full text screening) resulting in 111 total included citations. Image was created in Covidence software

A PRISMA flow diagram summarized the number of studies identified, excluded, and included (Fig. 1). We compiled counts and percentages of the number of references by type of journal publication (e.g., allergy/immunology vs. transfusion or transplantation vs. other academic topic) and citations included by research objective.

## Results

*Study period. *The scoping review was performed from November 21, 2024 – May 19, 2025.

*Screening*. Covidence software identified and imported 11732 studies for screening; 3858 studies were duplicates (3743 identified by Covidence, 115 duplicates manually removed).

**Fig. 2:**
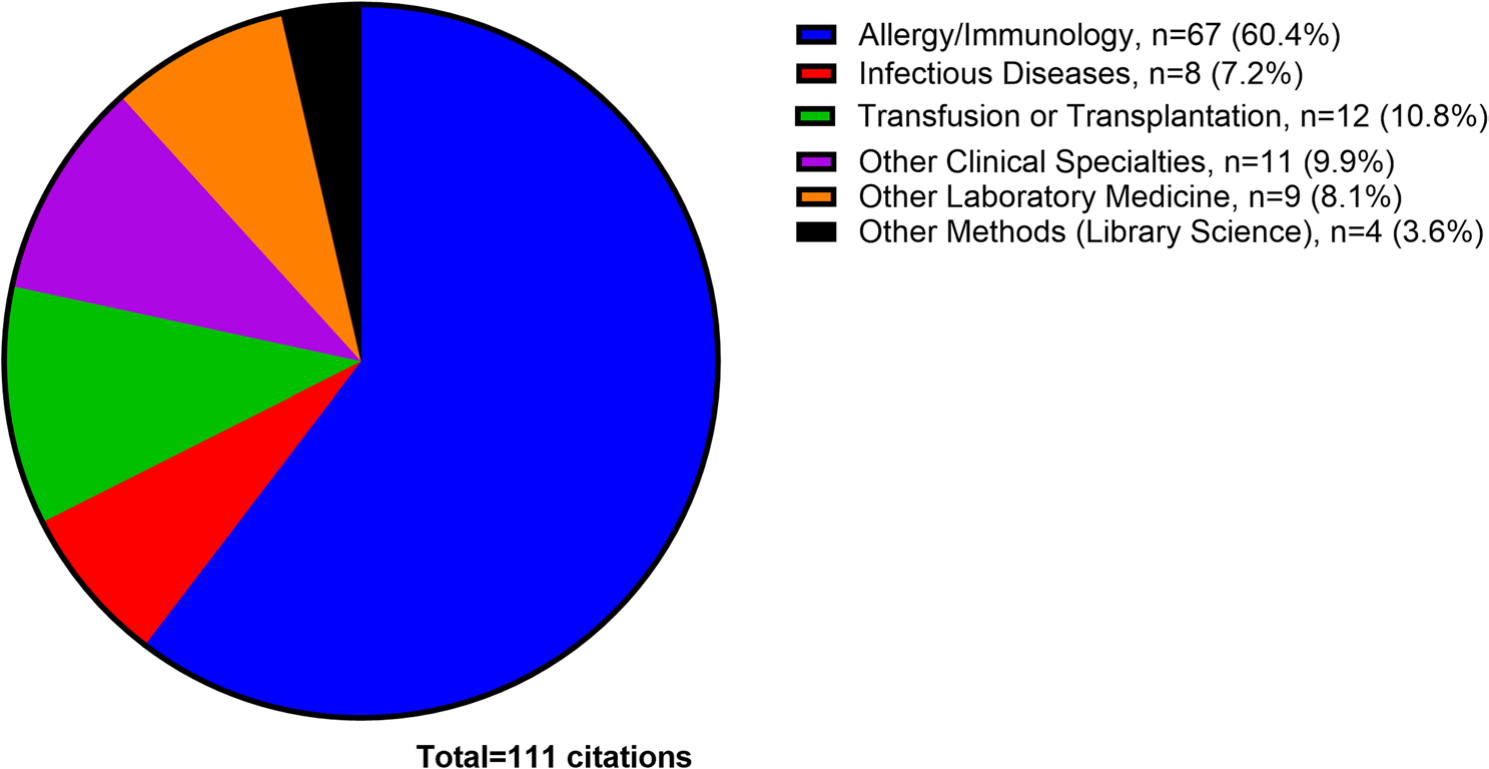
Topics of articles found in literature review (GraphPad). Counts and proportions of the 111 citations included in the literature review sorted by subject area of the journal where the research was originally published (e.g., *Journal of Allergy and Clinical Immunology* in “Allergy/Immunology” or *Transfusion* in “Transfusion or Transplantation”). Image was created in GraphPad

**Fig. 3:**
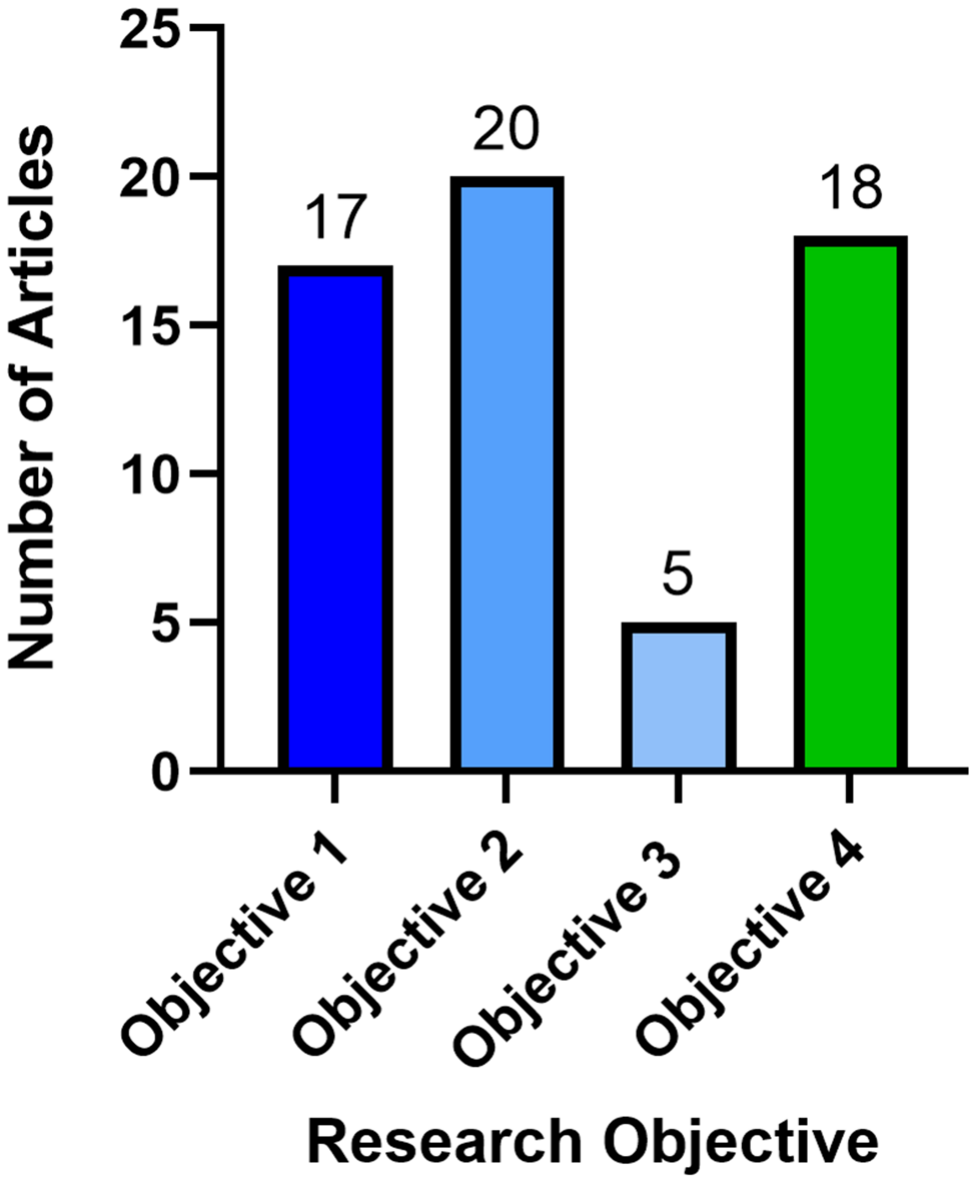
Citations included in literature review by research objective (GraphPad). Number of citations identified in the literature review which fulfilled research objectives. The main results cite 60 of 111 unique citations: from left to right, Objective 1 (*left)* described characteristics of all known cases of transfusion-related alpha-gal syndrome (TRAGS) (3 citations) and hypersensitivity reactions to infusions of mammalian red meat-derived medical products besides blood components that may resemble TRAGS (14 citations, for a total of 17 citations for objective 1). Objective 2 (*middle left*) identified studies that explore possible relationships between alpha-gal sensitization and blood group that may be relevant to understanding TRAGS (20 citations). Objective 3 (*middle right*) described which clinical, laboratory, and epidemiologic parameters used to diagnose AGS food allergy are also appropriate to diagnose TRAGS (5 citations, in addition to the 3 previously mentioned TRAGS case reports categorized under objective 1). Objective 4 (*right*) identified which diagnostic assays exist for AGS and how they are used for AGS and/or TRAGS, focusing on antibody and allergy testing (18 citations). Of the 111 total citations, 51 citations (not pictured) met research objectives but were moved to the introduction, methods, or discussion if they discussed aspects of the research topic better suited to those sections of the paper or had broader implications for research. Image was created in GraphPad

- *Title and abstract screening.* Of 7874 studies screened for eligibility, 7383 studies were excluded as irrelevant. One pair of reviewers agreed more than expected due to chance, and another pair disagreed more often than expected (Cohen’s kappa statistic = 0.68 and −0.01) (Additional Files, Supplemental Table 1).
- *Full text review.* Of 491 studies reviewed in full, 380 studies were excluded: irrelevant (309 studies), not found online (11 studies), not interpretable (5 studies), not reviewed by multiple reviewers (4 studies), duplicative (2 studies). Forty-nine review papers with no original research were excluded. Inter-rater reliability among all four reviewers was higher than expected (Cohen’s kappa statistic > 0) (Additional Files, Supplemental Table 2).
- *Data extraction.* After a final manual review for duplicative publications (e.g., poster presentations or preprints published before articles), 111 studies remained. The majority of the citations were published in allergy/immunology journals (67/111, 60.4%), with other citations appearing in transfusion or transplantation journals (12/111, 10.8%) or infectious diseases journals (8/111, 7.2%), among other specialties (Fig. 2). By research objective, 17 of 111 citations described characteristics of all known cases of transfusion-related alpha-gal syndrome (TRAGS) (3 citations) and hypersensitivity reactions to infusions of mammalian red meat-derived medical products besides blood components that may resemble TRAGS (14 citations) (objective 1); 20 citations identified studies that explore possible relationships between alpha-gal sensitization and blood group that may be relevant to understanding TRAGS (objective 2); 5 citations described which clinical, laboratory, and epidemiologic parameters used to diagnose AGS food allergy are also appropriate to diagnose TRAGS (not including the 3 TRAGS case reports already mentioned in objective 1) (objective 3); and 18 citations identified which diagnostic assays exist for AGS and how they are used for AGS and/or TRAGS (Fig. 3). Of the 111 total citations, 51 met research objectives but were moved to the introduction, methods, or discussion if they discussed aspects of the research topic better suited to those sections of the paper or had broader implications for research.
- *Grey literature search.* The first 250 hits in Google Scholar were screened from January 19, 2025 – February 27, 2025 using the search terms “alpha-gal” | “alpha-gal”) “blood”. No citations included randomized control trials or evidence-based guidance from professional societies. Thirty-two studies appeared in both the final list of 111 citations from the Covidence search and the first 250 studies in the grey literature. (Additional Files, Supplemental Material 4).

### Findings by research objective

#### Objective 1

The first objective was to describe characteristics of all known cases of transfusion-related alpha-gal syndrome (TRAGS) and hypersensitivity reactions to infusions of mammalian red meat-derived medical products besides blood components that may resemble TRAGS. Table 1 summarizes 5 total possible cases of TRAGS documented in 2022–2024 (Recipients #1-#5) and a sixth patient (Recipient #6) identified after the end of the study period. Recipient #1, a patient of unreported age and sex with group O blood, was undergoing a liver transplant in a metropolitan Washington, D.C. hospital in late 2022 when they had sudden onset of diffuse erythema and hypotension while receiving 1 unit of group B thawed plasma. Despite treatment including additional unspecified blood components, the patient died of coagulopathy. The recipient’s clinical risk factors for an ATR were the diffuse erythema and hypotension, with epidemiologic risk factors for anaphylaxis including a family history of severe cat allergy and a blood donor who lived with cats. Their laboratory risk factor for ATR was an elevated serum tryptase level (28.9 μg/L; ref ≤ 10.9 μg/L); serum anti-alpha-gal IgE levels were not measured because the clinicians did not yet know of AGS. This ATR was retrospectively characterized as a possible case of TRAGS after the clinicians encountered two other severe allergic reactions to group B blood in group O recipients (Recipients #2 and #3) over the next few months and learned of the region’s high prevalence of AGS [14]. Recipient #2, a 59-year-old man with group O blood with cirrhosis and bleeding from a duodenal ulcer, had a severe ATR to group B thawed plasma in February 2023. Clinical risk factors for this ATR were hypotension, hypoxia, and rash (with altered mental status potentially attributable to the patient’s underlying encephalopathy). Epidemiologic risk factors for anaphylaxis included a history of tick bites from work outdoors in parks as well as 42 previous transfusions, 3 of which were severe ATRs (2 to group B plasma, 1 to group O RBCs) requiring washed blood products to prevent ATRs. Laboratory risk factors were remarkable for serum anti-alpha-gal IgE level of 0.72 kU/L (ref < 0.1 kU/L). Supportive treatment (IV epinephrine, solumedrol, diphenhydramine) helped the patient recover from the ATR, but he remained encephalopathic from underlying liver disease. Recipient #3, a man of unspecified age with group O blood, sustained an intracranial hemorrhage in a fall 6 months after a living related donor kidney transplant for IgA nephropathy. He was transfused with group B psoralen-treated apheresis platelets for low platelet count ( < 100 k/µL) (thrombocytopenia), followed by several clinical risk factors for ATR including worsening mentation, whole body mottled erythema and hives, hypotension, and severe hypoxia requiring intubation. Epidemiologic risk factors for anaphylaxis were a possible history of tick bites, allergies to cats, dogs, horses, grass, and increased -ostomy bag output after consuming pork and beef. No past transfusion history was available. Laboratory risk factors were an anti-alpha-gal IgE level of 3.04 kU/L (elevated, but with no reference range provided). He survived the allergic event but developed *Klebsiella* pneumonia and post-transplant lymphoproliferative disorder after the intubation and died. Several months after these reactions, 2 nearby hospitals including ours recorded 2 more reactions with similar clinical, epidemiologic, and laboratory risk factors (Recipients #4 and #5). Recipient #4 was a 64-year-old man with group O blood seen in a different metropolitan D.C. area hospital in fall 2023 for acute COVID-19 mediated heart transplant rejection following a recent transplant for nonischemic dilated cardiomyopathy. He was on ECMO support. While undergoing plasma exchange (50% albumin/50% fresh frozen plasma) he tolerated several units of group O and A plasma before becoming hemodynamically unstable with airway edema and hypoxemia during transfusion of 1 unit of group B plasma, all clinical risk factors for ATR. There were no epidemiologic risk factors for anaphylaxis aside from him being in an area with high-prevalence for AGS, and his only known past transfusion reaction was 10 years before, an episode of transfusion-associated circulatory overload (TACO) to a unit of group AB plasma received while he was on a left ventricular assist device. Laboratory risk factors were an anti-alpha-gal IgE level of 1.96 kU/L (normal range ≤ 0.09 kU/L), mammalian red meat allergy panel positive for beef (0.39 kU/L) and negative for pork (0.21 kU/L) and lamb (0.20 kU/L) (normal range ≤ 0.34 kU/L for all meats) and serum tryptase of 54.1 ng/mL (normal range < 10.9 ng/mL). Plasma exchange was stopped. Epinephrine and norepinephrine drips were given for hypotension. The patient survived the ATR, then died several weeks later from sepsis. Recipient #5, seen at a different metropolitan D.C.-area hospital in fall 2023, was a 57-year-old man with group O blood who required resuscitation with red blood cells and plasma during an abdominal resection related to his history of melanoma and neurofibromatosis type 1. While receiving a group B plasma transfusion during this operation, he coded, with clinical risk factors for ATR including hives, hypotension, and ventricular tachycardia. Epidemiologic risk factors for anaphylaxis included geographic region and history of tick bites, but no past history of ATRs was available. Laboratory risk factors were notable for an anti-alpha-gal IgE level of 10.3 kU/L (ref < 0.10 kU/L) (no mammalian red meat allergy panel performed) and a normal serum tryptase level of 5.7 ng/mL (normal < 11.5 ng/mL). Resuscitation with CPR, epinephrine, diphenhydramine, and dexamethasone was successful, and the patient was alive as of 5 months after his operation [40, 46]. Recipient #6, seen at a hospital in New Hampshire, U.S.A. in 2025, is a 76-year-old man with group O blood who was undergoing coronary artery bypass graft surgery and aortic valve replacement who became hypotensive (100/62 to 28/24 mm Hg) after he received 50 mL of a unit of group B platelets. No bleeding was noted. Epidemiologic risk factors for anaphylaxis included a history of tick bites, though he had no other known allergies to drugs or meat and it is unknown whether he lived in a geographic area with a high prevalence of AGS. Laboratory risk factors were remarkable for an anti-alpha-gal IgE level of 1.49 kU/L that remained elevated 43 days later (0.65 kU/L) and elevated serum tryptase (27.4 mg/mL) measured 3 hours postoperatively. Additional allergy testing was performed, including serum IgA levels which were nondetectable on the day of the surgery and rebounded to 65 mg/dL (“near normal,” ref 70–400 mg/dL), negative prick and intradermal testing for rocuronium and protamine drugs used perioperatively, flow cytometry analysis showing high levels of B antigen on the transfused platelets as well as patient serum containing IgE-recognizing alpha-gal and indirect basophil activation tests on the patient’s plasma IgE demonstrating basophil activation upon stimulation with alpha-gal or B antigen. Treatment with 50 mg diphenhydramine normalized the patient’s blood pressure. He survived [47].

**Table 1:** Clinical, epidemiologic, and laboratory characteristics and clinical courses of six patients with transfusion-related alpha-gal syndrome, 2022–2025

	Parameters Relevant to Diagnosing Alpha-Gal Syndrome (AGS)			Clinical Course*	
Data Source	Clinical	Epidemiologic	Laboratory	Treatment	Outcome
Gilstad et al. (*Transfusion*, 2023) 10.1111/trf.17521 **#1**	*Time and Place* Late 2022 Metropolitan Washington, D.C. *Demographics* Sex not specified Age not specified *Transfusion History* *Recipient blood group*: O *Donor blood group*: B Severe hypotension, diffuse erythema, and accelerating coagulopathy during a liver transplant after administration of one unit of group B thawed plasma.	*Risk For Alpha-Gal Syndrome* *High-Prevalence Region*: Yes (D.C.) *Tick Bites*: Unknown *Allergies*: Family history of anaphylaxis to cats (case patient carried epinephrine auto-injector and received blood from donor later found to have lived with cats). *Risk for Allergic Transfusions* *Past transfusions*: 2 RBCs (no known transfusion reactions)	Serum alpha-Gal sIgE**: Not tested (clinicians not yet aware of AGS) *Mammalian Meat Allergy Panel*: None *Serum tryptase*: 28.9 μg/L [ref </= 10.9 μg/L] (taken 15 min after allergic reaction) *Intradermal prick testing*: None *Transfusion reaction*: Not reported	Resuscitation for blood pressure collapse and accelerating coagulopathy (regimen unspecified but included additional blood components of unspecified group)	Died (coagulopathy)
**#2**	*Time and Place* February 2023 Metropolitan Washington, D.C. *Demographics* Male 59 years *Transfusion History* *Recipient blood group*: O *Donor blood group*: B Cirrhosis and bleeding duodenal ulcer from alcohol use. Transfused with group B thawed plasma without pre-medication and experienced hypoxia, hypotension, rash.	*Risk for Alpha-Gal Syndrome* *High-Prevalence Region*: Yes (D.C.) *Tick Bites*: Yes (worked at parks) *Allergies*: None (may have collapsed after eating hamburger) *Risk for Allergic Transfusions* *Past transfusions*: 42 (3/42 severe allergic reactions, case patient required washed blood products) --*#1 (group O RBCs)*: rash, hypotension, hypoxia, altered mentation --*#2 (group B thawed plasma)*: chest pain, shortness of breath, rash despite pre-medication --*#3 (group B thawed plasma)*: hypoxia, hypotension, rash after administration (no pre-medication)	Serum alpha gal IgE**: 0.72 kU/L [ref < 0.1 kU/L] *Mammalian Meat Allergy Panel*: None *Serum tryptase*: None *Intradermal prick testing*: None *Transfusion reaction*: Not reported	Supportive (IV epinephrine, solumedrol, and diphenhydramine)	“Responded without consequences to [supportive] treatment” (but history was limited by case patient’s encephalopathy from underlying liver disease)
**#3**	*Time and Place* February/March 2023 Metropolitan Washington, D.C. *Demographics* Male Age not specified *Transfusion and Transplant History*: *Recipient Blood Group*: O *Donor Blood Group*: B Traumatic intracranial hemorrhage from a fall 6 months after receiving living related donor (sister) kidney transplant for IgA nephropathy and Crohn’s disease with diverting ileostomy (including one hospital admission for severe abdominal pain, nausea and vomiting). During transfusion of a unit of group B psoralen-treated apheresis platelets for count <100 K/μL, acute worsening of mentation, whole body mottled erythema and hives, hypotension, severe hypoxia requiring intubation.	*Risk for Alpha-Gal Syndrome* *High-Prevalence Region*: Yes (D.C.) *Tick Bites*: Possible (per sister) *Allergies*: cats, dogs, horses, grass; increased -ostomy bag output possibly after consuming pork, beef *Risk for Allergic Transfusions* *Past transfusions*: None	Serum alpha gal IgE**: 3.04 kU/L [no reference range provided] *Mammalian Meat Allergy Panel*: None *Serum tryptase*: None *Serum IgA*: Within normal limits *Intradermal prick testing*: None *Transfusion reaction*: Not reported *Post-transfusion blood culture*: Negative	Not specified (developed *Klebsiella* pneumonia shortly after intubation)	Died 25 days after allergic event (underlying post-transplant lymphoproliferative disorder)
Miller et al. (*Transfusion*, 2024) 10.1111/trf.17811 **#4**	*Time and Place* Fall 2023 Metropolitan Washington, D.C. *Demographics* Male 64 years *Transfusion History* *Recipient Blood Group*: O *Donor Blood Group*: B Status post heart transplant for nonischemic dilated cardiomyopathy with acute COVID-19 induced antibody-mediated transplant rejection. On ECMO support. While undergoing plasma exchange (50% albumin/50% fresh frozen plasma), tolerated 2 units of group O plasma and 1 unit group A plasma before becoming hemodynamically unstable with airway edema, hypoxemia during transfusion of 1 unit group B plasma.	*Risk for Alpha-Gal Syndrome* *High-Prevalence Location*: Yes (D.C.) *Tick Bites*: None known *Allergies*: None known *Risk for Allergic Transfusions* *Past transfusions*: Transfusion-associated circulatory overload (TACO) reaction to group AB plasma 10 years before heart transplant while on left ventricular assist device. No other known allergic, anaphylactic, or anaphylactoid transfusion reactions from plasma transfusions. (chest X-ray excluded transfusion-related acute lung injury (TRALI))	Serum Alpha-Gal IgE**: 1.96 kU/L [normal range ≤ 0.09 kU/L] *Mammalian Meat Allergy Panel* *Pork*: 0.21 kU/L [normal range ≤ 0.34 kU/L] *Beef*: 0.39 kU/L [normal range ≤ 0.34 kU/L] *Lamb*: 0.20 kU/L [normal range ≤ 0.34 kU/L] *Serum tryptase*: 54.1 ng/mL [normal range < 10.9 ng/mL] (collection time not available) *Intradermal prick testing*: None *Transfusion reaction*: *Antibody screen*: negative *Direct antiglobulin test (DAT)*: Negative	Plasma exchange was stopped. Epinephrine, norepinephrine drips for hypotension from acute tachycardia and diastolic hypotension.	Died several weeks later (sepsis from underlying illness)
**#5**	*Time and Place* Fall 2023 Metropolitan Washington D.C. *Demographics* Male 57 years *Transfusion History* *Recipient Blood Group: O* *Donor Blood Group: B* Abdominal resection related to melanoma, neurofibromatosis type 1 involved resuscitation with 3 units of group O RBC and 2 units of group B plasma, with the group B plasma transfusion leading to hives, hypotension, and ventricular tachycardia during an intraoperative code.	*Risk for Alpha-Gal Syndrome* *High-Prevalence Location*: Yes (D.C.) *Tick Bites*: Yes *Allergies*: None known *Risk for Allergic Transfusions*: None known (chest X-ray excluded transfusion-related acute lung injury (TRALI))	Serum Alpha-Gal IgE**: 10.3 kU/L [ref <0.10 kU/L] *Mammalian Meat Allergy Panel*: Not performed *Serum Tryptase*: 5.7 ng/mL [normal < 11.5 ng/mL] (collected within 30 min of reaction) *Intradermal prick testing*: None *Transfusion reaction*: *Antibody screen*: negative *Direct antiglobulin test (DAT)*: Negative	CPR Administration of epinephrine, diphenhydramine, dexamethasone	Return of spontaneous circulation achieved following resuscitation Alive as of 5 months after reaction
Jones et al. (*Transfusion*, 2025) 10.1111/trf.18405 **#6**	*Time and Place* Date not specified (2024?) New Hampshire *Demographics* Male 76 years *Transfusion History* *Recipient Blood Group*: O *Donor Blood Group*: B While undergoing coronary artery bypass graft (CABG) surgery, the case patient became severely hypotensive after receiving ~50 mL of a group B platelet unit.	*Risk for Alpha-Gal Syndrome* *High-Prevalence Location*: Possibly (New Hampshire has medium to high prevalence, patient’s travel unknown) *Tick Bites*: Yes *Allergies*: None known *Risk for Allergic Transfusions*: None known	Serum alpha-gal IgE**: 1.49 kU/L [ref <0.10 kU/L] (day of surgery) 0.65 kU/L (43 days post-op) *Mammalian Meat Allergy Panel*: none *Serum tryptase*: 27.4 ng/mL [ref ≤8.4 ng/mL] (3 h post-op) *Serum IgA (mg/dL)*: 65 mg/dL [ref 70–400 mg/dL] (2 days post-op) *Intradermal prick testing*: --Rocuronium (negative) --Protamine (negative) *Transfusion reaction*: Not reported *Flow cytometric analyses*: 1) Transfused platelets expressed B antigen 2) Patient’s plasma contained IgE-recognizing alpha-gal and B antigen > A antigen *Indirect basophilic activation test*: resting allogeneic basophils incubated with patient’s plasma showed equivalent (weak) activation when stimulated with alpha-gal or B antigen.	Transfusion was stopped Vasopressors (epinephrine, methylene blue, vasopressin) 50 mg diphenhydramine	Blood pressure normalized after stopping transfusion and administering diphenhydramine. Otherwise normal postoperative course Discharge home postoperative day #4

*Only severe TRAGS reactions have been reported; CDC National Healthcare Safety Network (NHSN) hemovigilance guidelines do not mandate reporting non-severe allergic transfusion reactions such as itching.

**Reference ranges for anti-alpha-Gal IgE may vary. In general, a result > 0.1 kU/L on the ThermoFisher Phadia ImmunoCAP assay may be considered positive for alpha-Gal sensitization.

Table 1 describes all TRAGS patients in detail, with the caveat that less severe TRAGS cases could have been missed in other people with clinical, epidemiologic, or laboratory evidence of alpha-gal sensitization [48] because not all U.S. hospitals mandate reporting non-severe ATRs (itching, hives, etc.).

Because this case series had limited information on diagnosis and management of TRAGS, we reviewed how other clinicians managed hypersensitivity reactions to infusions of mammalian red meat-derived medical products besides blood components. Potential triggers of hypersensitivity reactions in alpha-gal sensitized people include cetuximab (a chimeric mouse-human monoclonal antibody biologic that targets the epidermal growth factor receptor and contains the alpha-gal oligosaccharide present on the mouse-derived Fab portion of the cetuximab heavy chain) [49, 50]), unfractionated and low-molecular weight heparin (sourced from porcine intestinal mucosa) [51, 52], bovine gelatin-based colloid plasma volume substitutes [53, 54], bovine thrombin [55], and other mammalian red meat-derived biologics [56–58]. It is unknown which people who experience hypersensitivity reactions to these substances may also be at risk for TRAGS, but allergists include AGS on a differential diagnosis of idiopathic anaphylaxis [59–61]. Successful management of these infusion reactions includes trigger avoidance (and/or measuring anti-alpha-gal IgE levels to triage which patients need to avoid triggers, as was successfully tried in a cohort of 16/206 patients in Japan eligible for cetuximab therapy for squamous cell carcinoma of the head and neck [62]). Standardized clinical outcomes and epidemiologic and laboratory measures are needed in future case series of TRAGS to create a case definition that could guide treatment and trigger avoidance (e.g., avoiding transfusions with group B plasma).

#### Objective 2

The second objective was to identify studies that explore possible relationships between alpha-gal sensitization and blood group that may be relevant to understanding TRAGS. Because there were no other publications on TRAGS, we reviewed the literature on alpha-gal sensitization, AGS, and blood group. In the early 20^th^ century, Karl Landsteiner found, within anti-B sera, an antibody to a “B-like antigen” later identified to be the alpha-gal antigen. Galili et al. found in the 1980s that the specificity of anti-alpha-gal antibodies may depend on blood group: a proportion of alpha-gal antibody clones cross-reacted with the B antigenic determinant due to lack of immune tolerance in non-group B or AB individuals in whom the B antigen is absent [63]. A decade later, McMorrow et al. found in a small sample of human sera from an academic hospital in the northern United States that B antigen-expressing donors (people with group B or AB blood) had decreased reactivity to alpha-gal compared to people who do not express the B antigen (people with A or O blood) [64], a finding later tested by clinical epidemiologists.

During the 2000s-2020s, clinicians began to identify patients with severe allergic reactions to mammalian red meat and red meat-derived products that were linked to alpha-gal sensitization. Some cohort and case-control studies examining alpha-gal and the immune response in such patients collected data on blood group to test whether there was an association between clinical, epidemiologic, and/or laboratory risk factors for alpha-gal sensitization and blood group. The only systematic review of them, a meta-analysis of 92 patients with mammalian red meat allergy from 4 institutions (U.S., Spain, Sweden, Japan) published by Brestoff et al. in 2018, found that individuals with group B blood were 5 times less likely to be afflicted with a mammalian red meat allergy than those with group O blood [65]. Several subsequent publications reached similar conclusions about an association between blood group and alpha-gal sensitization, including one large cohort study on people in a high-prevalence area of AGS in the southern United States (North Carolina) in which people with group B blood were one-fourth as likely to have AGS as people of other blood groups [66]. In several other cohorts, group B individuals had lower IgE anti-alpha-gal antibodies than individuals of other blood groups [66–71]. The largest of these studies, from a longitudinal cohort from Denmark, showed that people with group B blood had partial protection from alpha-gal sensitization (as measured by an anti-alpha-gal IgE titer ≥ 0.1 kU/L). The way blood group was tested is unlikely to be a contributing factor: Blood group in the Danish study was determined by next-generation sequencing (Illumina Omniexpress chip testing), a test that has > 99% accuracy for identifying ABO group but is not a routine clinical typing method used by blood banks [20]. A cross-sectional study by Cabezas-Cruz et al. used isoagglutinin testing, another non-routine typing method with lower sensitivity and specificity than serology or NGS, and their use of this method did not diminish their conclusion that reduced susceptibility to alpha-gal sensitization in individuals with group B blood in their cohort potentially contradicted the evidence for the B antigen’s “protective effect” [72].

Despite this evidence, results from some of the same areas of the U.S. and Europe with a high prevalence of AGS feature conflicting findings on alpha-gal and blood group such as the finding documented by Cabezas-Cruz et al. [72]. Beyond their study, blood group B has been shown to be only partly protective against mammalian red meat allergy in people, as people with the B antigen can still acquire clinical manifestations of alpha-gal sensitization or even AGS [73]. Thus, this hypothesized group B/AB “protective effect” [35, 71] has not been consistently observed [74]. The question at this point is: which factor(s) contribute to the discrepant results? Two possible contributing factors stood out. First, could the methods used to calculate the prevalence of blood groups containing the B antigen in small cohorts versus the general population from which these cohorts are sampled have contributed? For example, elevated levels of anti-alpha-gal IgE have been observed in individuals with group B and AB blood in some cohorts, but the percentage of people with group B blood may be underrepresented in these samples relative to the distribution of people with group B blood in their country. Countries may have different proportions of group B blood in the population and in the sub-population of people with AGS: for example, < 2% of people in a cohort of AGS patients from Sweden had the B antigen in their blood [18], however a later Swedish cohort showed 5.9% frequency of the B antigen (including groups B and AB), a number that more closely reflects the frequency of people with group B blood in the Swedish population [35]. A comparable cohort of people with AGS from the southern United States had a higher proportion of people with group B blood (8%) than in the Swedish cohorts, close to the expected percentage of people with group B blood in the Caucasian population, but this percentage may not reflect the true distribution of group B or AB blood in the larger regional population [73]. These studies may have recruited fewer people with group B or AB blood into their studies than existed in their source populations, meaning more information on clinical, laboratory, and epidemiologic risk factors for alpha-gal sensitization in people with group B or AB blood may yet to be collected. Second, could the different ways that various studies defined and measured alpha-gal sensitization or AGS as a clinical, laboratory, or epidemiologic outcome impact the results? Some studies defined people with AGS as anyone who provided a blood sample that tested positive for anti-alpha-gal IgE (usually defined as > 0.1 kU/L, the positive cutoff of the gold standard ImmunoCAP assay). Some researchers of populations with a high prevalence of AGS [17] or high risk for severe allergy (e.g., recurrent idiopathic anaphylaxis [59]) used higher titer levels as positive cutoff values. Other studies defined people with AGS as those with a laboratory risk factor for AGS (blood samples positive for anti-alpha-gal IgE) and one or more clinical and epidemiologic risk factors for red meat allergy diagnosed by an allergist, based on responses to a questionnaire covering clinical symptoms, diet, allergies, or other epidemiologic exposures. Different researchers used different questionnaires depending on their study population (e.g., allergy clinic versus occupational studies of people who work outdoors [69, 75]). Though almost all these studies claimed that having group B or AB blood was protective against AGS, the authors defined who had AGS or not differently based on the best evidence they had at the time. Unfortunately, the current evidence does not clearly indicate higher rates in individuals with group O or A blood compared with individuals with group B blood, despite theoretical tolerance differences. The notion that B or AB individuals are “protected” remains inconsistent across studies. No systematic studies have assessed transfusion outcomes in recipients stratified by both blood group and alpha-gal sensitization. Clarifying whether this “protective effect” exists and how it is measured would refine interpretation of ABO-linked hypotheses, as several investigators of alpha-gal and the immune response have encouraged [76].

#### Objective 3

The third objective was to determine which clinical, laboratory, and epidemiologic parameters used to diagnose AGS food allergy are also appropriate to diagnose TRAGS. No original research addressed this question, but multiple authors found it important. Diagnostic biomarkers of AGS beyond anti-alpha-gal IgE levels may be needed [33, 38]. Investigators are beginning to look beyond standard clinical and laboratory parameters of AGS and TRAGS to find future possible biomarkers of AGS and TRAGS. One biomarker of interest, interleukin-4 (a Th2 cytokine that promotes class-switching), was required for alpha-gal sensitization in a mouse model. IgE and IgG1 levels were depleted in mice without IL-4. Therapies targeting IL-4 production in humans (e.g., dupilumab, a monoclonal antibody targeting the alpha-subunit of the IL-4 receptor currently used to treat allergic conditions including asthma and eczema) could hypothetically reduce symptoms of alpha-gal sensitization, but no one has tested this off-label use in clinical trials [77]. Other research on alpha-gal sensitization includes characterization of the T cell response (in which memory B cells proliferated in healthy volunteers who were exposed to alpha-gal or the B antigen) [78] and skin-infiltrating basophil response (in which Th2 polarization by basophils led to anti-alpha-gal IgE antibody production in volunteers with multiple tick bites) [31], but none of this work has direct clinical applications yet. It is unknown which aspects of this research may one day help characterize AGS or TRAGS. Given these gaps, the next step was to examine the most common markers in the reported AGS cases. Reviewing these common markers may be a good starting point in linking experimental findings to clinical presentation.

Common clinical risk factors in all 6 cases described in Objective 1 were chronically ill men aged 50–80 years with group O blood who experienced hypotension (6/6 patients) after transfusion of a group B blood product (plasma in 4 patients, a psoralen-treated apheresis platelet unit in 1 patient, an apheresis platelet with no information about pathogen reduction or additive solution in 1 patient) during or after major surgery (3 solid organ transplant patients, 2 patients in abdominal surgery, 1 patient receiving coronary artery bypass graft and valve replacement) without a definitive diagnosis or exposure source for their ATR. Common laboratory risk factors for these ATRs were above-normal serum tryptase levels post-transfusion (3 patients, plus 1 patient with a normal tryptase level who may not have been tested immediately after the ATR) and anti-alpha-gal IgE levels above 0.1 kU/L (5 of 5 patients measured). Common epidemiologic risk factors for ATR were tick bites (4 patients). One patient had past ATRs (Table 2). For treatment, the patients received supportive care (e.g., antihistamines, IV steroids, restriction of group B plasma-containing products as trigger avoidance). Outcomes varied: though 4 patients survived the ATRs, 3 patients died (1 from coagulopathy after the ATR, 2 from underlying conditions several weeks after the ATR) (Additional Files, Supplemental Table 3).

**Table 2:** Proposed provisional case definition of transfusion-related alpha-gal syndrome (TRAGS)

	Case#1	Case#2	Case#3	Case#4	Case#5	Case#6
**Reason for Hospital Admission**	liver transplant	bleeding duodenal ulcer (alcohol-related cirrhosis)	traumatic intracranial hemorrhage (ICH); IgA nephropathy, Crohn’s disease with diverting ileostomy	acute COVID-19 induced antibody-mediated heart transplant rejection on ECMO	abdominal resection (history of melanoma and neurofibromatosis type 1)	coronary artery bypass graft surgery and aortic valve replacement
Clinical Criteria*						
**Hypotension**	**Y**	**Y**	**Y**	**Y**	**Y**	**Y**
Altered mental status		**Y**	**Y**			
Erythema	**Y**		**Y**			
Hives			**Y**		**Y**	
Hypoxia		**Y**	**Y**			
Coagulopathy/Thrombocytopenia	**Y**		**Y**			
Angioedema				**Y**		
Dyspnea				**Y**		
Rash		**Y**				
Tachycardia				**Y**	**Y**	
Hypoxemia				**Y**		
Laboratory Criteria (Reactivity)**						
**Serum or plasma sIgE**		**Y**	**Y**	**Y**	**Y**	**Y**
**Serum tryptase**	**Y**			**Y**	N	**Y**
Serum immunoglobulins (other than IgE)			N			N
Pork IgE				N		
Beef IgE				**Y**		
Lamb IgE				N		
Intradermal prick testing for 1+ allergens						**Y**
Flow cytometry for B antigen						N
Flow cytometry for alpha-Gal IgE						**Y**
Indirect basophil activation test						**Y**
Epidemiologic Criteria***						
**Group O recipient of B plasma-containing product from donor in area with medium/high AGS prevalence**	**Y**	**Y**	**Y**	**Y**	**Y**	**Y**
**History of tick bite(s)**		**Y**	**Y**		**Y**	**Y**
**Residence or time outdoors in a region defined by CDC as high AGS prevalence**				**Y**	**Y**	
History of mammalian meat allergy					N	N
Past allergic transfusion reactions	N	**Y**	N	N	N	N
Other allergies	**Y**	N	**Y**		N	N

Bold indicates the most represented factors/results

*blank cell = not reported

**blank cell = not tested

***blank cell = unknown

N = No

Y = Yes

#### Objective 4

The fourth objective was to identify which diagnostic assays exist for AGS and how they are used for AGS and/or TRAGS. Sensitive, precise immunoassays can help diagnose AGS, with potential for cross-reactivity [79–81] and imprecise correlations between test results and clinical symptoms of alpha-gal sensitization [82]. Brestoff et al. conducted a systematic review to determine the sensitivity, specificity, and predictive value of IgE tests for meat allergy, finding data on 135 patients and 37 controls showing strongest performance by serum alpha-gal bovine thyroglobulin (b-TG) IgE (sensitivity 100% for detection of red meat allergy). In these patients total IgE had a lower positive predictive value (PPV) than tests to discriminate allergy to beef, lamb, or pork and could not distinguish exposures to ticks or cetuximab [83].

The performance of the ImmunoCAP solid phase anti-alpha-gal antibody specific IgE testing, the clinical gold standard for measuring alpha-gal IgE, most interests clinical researchers [84, 85], but it may not be the best test for every institution; before the ImmunoCAP became widely used in clinical practice, researchers experimented with enzyme-linked immunosorbent assays (ELISA) to measure anti-alpha-gal antibody levels [86] as well as a time-resolved immunofluorometric assay [67], but ELISA testing has lower sensitivity than the ImmunoCAP due to reduced interference from competing natural alpha-gal specific IgM, IgG, and IgA antibodies. Since then, Li et al. and others have studied the possibility of developing a competition ELISA [87], as individuals with AGS may have IgM and IgG positivity against alpha-gal before their diagnosis but only develop IgE levels later. Chakrapani et al. reviewed the allergenicity of glycolipids and glycoproteins in vitro for biomarkers of metabolic activity [88]. Outside the studies in this review, it has been shown that patients with anaphylaxis may have stable or undetectable IgE levels [89, 90], raising concern about the utility of IgE as biomarker to predict severe allergy. Food allergy researchers already recognize that IgE levels may not necessarily correlate with the severity of allergy and thus may not be useful screening tools. The anti-alpha-gal IgE diagnostic assay has been reported to produce false positives due to other sources of IgE sensitization [89, 91]. The basophilic activation test (BAT) may differentiate between patients with asymptomatic alpha-gal sensitization and AGS [92]. Apostolovic et al. studied sera from 3 patients allergic to red meat and 1 healthy volunteer with no allergy history to investigate if the BAT or alpha-gal-specific IgE and IgG1 antibody avidity suggested clinical phenotypes of red meat allergy. In this study, basophil reactivity seemed to be higher in the meat-allergic patients, but neither anti-alpha-gal IgE nor IgG1 avidity could distinguish the severity of allergic reaction [9]. Serum tryptase measurements may not be readily available or necessary in emergency diagnoses [93].

These assay performance studies did generally address the proposed association between B blood group and susceptibility to alpha-gal-related allergy [94]. Buonomano et al., who conducted an early ELISA serosurvey of anti-alpha-gal IgM and IgG antibodies in 200 healthy people, reported the distribution of blood groups in their study sample because contemporary xenotransplant studies showed that at least 1% of people with B antigen may produce anti-alpha-gal antibodies [95]. Yet comparatively few studies report blood group data. An analysis of healthy blood donors in Denmark published in 2011 controlled for blood group [67], showing lowest levels of anti-alpha-gal IgG antibodies in individuals expressing the B antigen and slightly higher levels in an A “intermediate” group. Testing patients with unexplained severe transfusion reactions who have a history of AGS or tick exposure for alpha-gal specific IgE may be the most appropriate recommendation to investigate TRAGS to date.

**Fig. 4:**
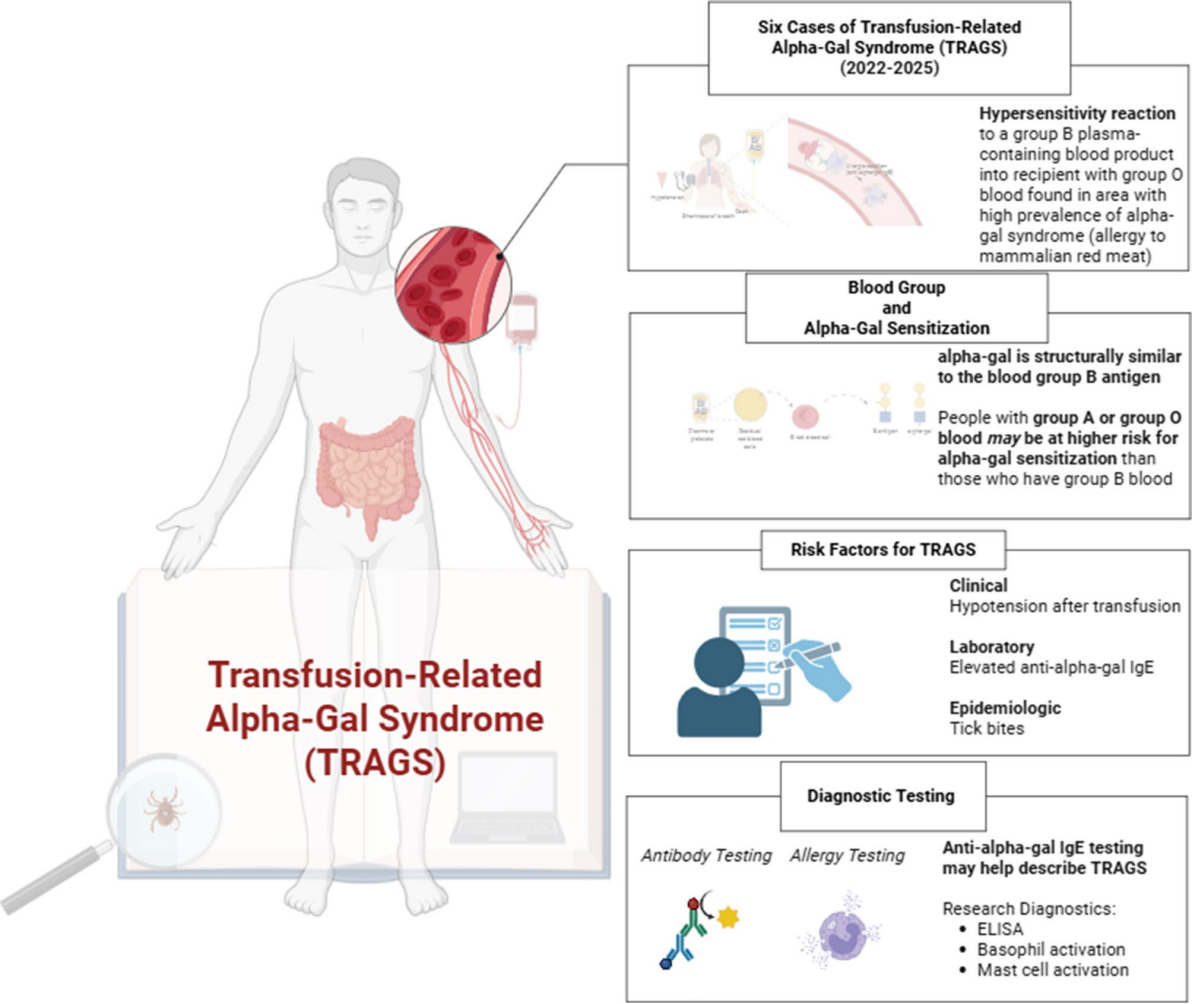
Graphical summary of literature review results (Biorender). This graphical summary summarizes the main results of the scoping review of the literature on transfusion-related alpha-gal syndrome (TRAGS) (*lower left*). Objective 1 (“Six Cases of Transfusion-Related Alpha-Gal Syndrome (TRAGS) (2022–2025)”) (*upper right*) described characteristics of all known cases of transfusion-related alpha-gal syndrome (TRAGS) and hypersensitivity reactions to infusions of mammalian red meat-derived medical products besides blood components that may resemble TRAGS. Objective 2 (“Blood Group and Alpha-Gal Sensitization”) (*middle upper right*) identified studies that explore possible relationships between alpha-Gal sensitization and blood group that may be relevant to understanding TRAGS. Objective 3 (“Risk Factors for TRAGS”) (*middle lower right*) described which clinical, laboratory, and epidemiologic parameters used to diagnose AGS food allergy are also appropriate to diagnose TRAGS. Objective 4 (“Diagnostic Testing”) (*lower right*) identifies which diagnostic assays exist for AGS and how they are used for AGS and/or TRAGS. Image was created in Biorender

Findings for all four research objectives are summarized in a graphical summary (Fig. 4).

## Discussion

This scoping review of the literature on alpha-gal sensitization and allergic transfusion reactions found important points that may inform clinicians’ understanding of TRAGS. Clinical, epidemiologic, and laboratory risk factors associated with all known cases of TRAGS include rash, shortness of breath, and/or hypotension in group O recipients of group B plasma-containing products who had elevated serum tryptase and anti-alpha-gal sIgE levels found outside the traditional work-up for a transfusion reaction, but only severe reactions (e.g., anaphylaxis) were typically reported, reflecting a narrow range of AGS-like reactions found in regions with a medium to high prevalence of AGS that may not be representative of the wide spectrum of AGS reactions observed in allergy clinics worldwide. Foregoing administration of allergens has been the first line of prevention of anaphylaxis in alpha-gal sensitized patients, but the TRAGS case reports lack dietary challenge and avoidance data. Possible relationships between alpha-gal sensitization and blood group that may be relevant to TRAGS have been identified in bench research, but the current evidence does not clearly indicate higher rates in O or A individuals compared with B, despite theoretical tolerance differences. The notion that B individuals or AB individuals are “protected” remains inconsistent across studies, with conflicting results across population cohorts (e.g., Denmark vs. U.S.). No systematic studies have assessed transfusion outcomes in recipients stratified by both blood group and alpha-gal sensitization. Parameters used to diagnose AGS may be useful for diagnosing transfused patients with suspected TRAGS, but more original research is needed. Diagnostic biomarkers of AGS beyond anti-alpha-gal sIgE levels are under research to better correlate clinical symptoms of AGS and test results. Overall, these results may raise more questions than they answer about diagnosing, managing, and preventing TRAGS, but clinicians and researchers may find them useful as more is learned about this newly recognized condition.

This is the first comprehensive review of AGS applicable to hematology, blood banking, allergy/immunology, and laboratory science. It documents a possible association between alpha-gal sensitization and blood group. Importantly, these data do not definitively correlate blood group to the clinical diagnosis of AGS. Many alpha-gal sensitized individuals in the community could theoretically react to alpha-gal bearing products but do not have symptomatic AGS or experience anaphylaxis following consumption of mammalian-derived red meat products, so the true clinical risk of TRAGS remains unknown. Such caveats are not uncommon in allergy or transfusion medicine research: for example, researchers experienced similar challenges in evaluating the possible evidence base for another suspected etiology of transfusion-related anaphylaxis, IgA deficiency [96].

Several significant studies of AGS that may be relevant to TRAGS were published after our study period. The most read of these publications was a clinical case report of the first autopsy-confirmed death of an otherwise healthy 47-year-old man from alpha-gal related anaphylaxis 4 hours after experiencing gastrointestinal symptoms followed by anaphylaxis after exposure to red meat while camping. This decedent was confirmed to have laboratory evidence of alpha-gal sensitization on a postmortem evaluation (anti-alpha-gal sIgE 0.57 IU/mL) [97]. Other research included previously unreported findings implicating *Ixodes* spp. as risk factors for AGS [98, 99] (raising concern for co-infection or multiple tickborne illnesses), new data from a longitudinal cohort of AGS patients in the southeastern U.S. showing increased anti-alpha-gal IgG levels in subjects with blood groups A and O [100], and hemovigilance data from France showing an increased risk of severe ATRs in group O recipients of group B or AB plasma or platelet concentrate from January 1, 2022 to December 31, 2024 [101]. Most significantly for researchers of TRAGS, Dunbar et al. published preliminary results from a multi-site study that estimated risk for TRAGS at 14 participating sites in 10 countries during a 2-year period [41]. Based on their and our work, we have tentatively outlined a proposed case definition for TRAGS. This includes provisional considerations for laboratory evaluation and potential trigger avoidance. Recently Ladowski et al. proposed pre-screening blood products prior to human xenotransplant [102], but Dunbar et al. noted in their consideration of the costs and benefits of pre-screening that risk reduction strategies such as prohibiting Group O patients from receiving B or AB plasma and platelets may be resource-intensive. The results of this review also suggest that such broad risk-reduction strategies may not yet be justified except for individual patients with AGS-like symptoms of anaphylaxis.

Because our research questions focused on blood group, the review excluded papers on AGS diagnostics without blood group data. Pre-clinical animal model studies continue to examine alpha-gal activity [103]. This literature described other diagnostic methods that could be employed in the future, including the mast cell activation test (MAT), histamine release (HR) assay, and -omics technologies. The MAT is a promising in vitro diagnostic tool for AGS, measuring CD63 expression to detect mast cell degranulation in response to allergen-specific IgE. Compared to the BAT it offers greater sensitivity and the option to use frozen serum samples, facilitating logistics and sample handling [104, 105]. The HR assay is another emerging test that evaluates histamine release from activated basophils via fluorescence, possibly allowing differentiation of AGS from sensitization to alpha-gal. Further validation is needed before this could be a diagnostic tool [106]. Omics technologies could be a powerful tool to identify diagnostic biomarkers, but their complexity and resource requirement leave these techniques in the research setting [107–109]. Correlation between laboratory results and clinical history is recommended, even among those who study IgE cross-reactivity immune profiling in people with AGS [110, 111]. Combining the best supported assays for AGS and a targeted transfusion medicine evaluation may be the best approach currently, considering the lack of a validated diagnostic algorithm for TRAGS.

## Conclusions

In conclusion, this scoping review has all known information on blood group and potentially fatal transfusion reactions known as transfusion-related alpha-gal syndrome (TRAGS). It may assist clinicians in evaluating severe allergic transfusion reactions while blood banking and public health organizations create evidence-based diagnostic and management guidelines for TRAGS. The true incidence of alpha-gal-related transfusion reactions, including mild or unrecognized cases, is likely underreported and warrants further study. We suggest that practitioners collect complete allergy histories and anti-alpha-gal laboratory testing and practice caution when administering out-of-group plasma-based products or products derived from mammalian red meat in the transfusion and transplant settings. To close the experimental gap, future systematic studies could assess transfusion outcomes in recipients, stratified by both blood group and alpha-gal sensitization. Clarifying how transfusion outcomes in recipients are measured and compared would refine interpretation of ABO-linked hypotheses. As alpha-gal sensitization becomes more widespread, clinical guidance about the potential for severe morbidity and mortality from severe allergy from AGS is needed.

## Electronic supplementary material

Below is the link to the electronic supplementary material.

Supplementary Material 1

Supplementary Material 2

Supplementary Material 3

Supplementary Material 4

Supplementary Material 5

Supplementary Material 6

Supplementary Material 7

## Data Availability

The data generated and analyzed during the current study are available from the Covidence software repository “alpha-gal syndrome (CC)” [https://app.covidence.org/reviews/499113]. Restrictions may apply to the availability of these data, which were used under license for the current study via a library subscription and may not be publicly available. All scientific and grey literature in this review are publicly available on PubMed or in Google Scholar databases, and our search strings are in the Additional Files. Additional data are available from the authors upon reasonable request and with permission of the U.S. National Institutes of Health and Covidence.

## Abbreviations

alpha-gal (alpha-Gal): Galactose-alpha-1,3-Galactose (Gal-alpha1-3 Galβ1-(3)4GlcNAc-R)
ATR(s): allergic or anaphylactic transfusion reaction(s)
NIH CC: U.S. National Institutes of Health Clinical Center
TRAGS: transfusion-related alpha-gal syndrome
