# Supplementary material for: Alpha-gal sensitization and allergic blood transfusion reactions: a scoping review
Source: J Transl Med. 2026 Feb 4;24:325. doi: 10.1186/s12967-025-07614-9 (PMC12964899; doi:10.1186/s12967-025-07614-9)
Supplement: Supplementary file 2 — Supplementary Material 2 [file 12967_2025_7614_MOESM2_ESM.docx]

Supplemental Material: Search Strategies

| Review Title | Alpha-gal sensitization as a potential mechanism of allergic transfusion reactions to B OR AB type blood products: a scoping review |
| --- | --- |
| Databases Searched | PubMed (NCBI), Embase (Embase.com), Cochrane CDSR and CENTRAL (Wiley) Scopus |
| Date of Search | November 13, 2024 |
| Limits/Filters | None |
| Key Articles | provided |
| Search Created and Performed By | Carrie Price, MLS, Tracy Shields, MSIS, Biomedical Librarians, NIH Library, Office of Research Services, OD, National Institutes of Health |
| Total Number of Results | 11,732 |
| Results by Database | PubMed: 1,195  Embase: 1,946  Cochrane CENTRAL: 29  Web of Science: 4,586  Scopus: 3,976 |
| Duplicates | 3,743 identified by Covidence |
| Remaining Results to Screen | 7,989 |
| Update Date | n/a |

# PubMed (NCBI)

| **Set** | **Concept** | **Search Strategy** | **Results** |
| --- | --- | --- | --- |
| #1 | red meat allergy | ("red meat allergy"[nm] OR "mammalian meat allergy"[tiab:~6] OR "mammalian meat sensitivity"[tiab:~6] OR "mammalian meat hypersensitivity"[tiab:~6] OR "mammalian meat hyper sensitivity"[tiab:~6] OR "red meat allergy"[tiab:~6] OR "red meat sensitivity"[tiab:~6] OR "red meat hypersensitivity"[tiab:~6] OR "red meat hyper sensitivity"[tiab:~6] OR "mammalian meat allergies"[tiab:~6] OR "mammalian meat sensitivities"[tiab:~6] OR "mammalian meat hypersensitivities"[tiab:~6] OR "mammalian meat hyper sensitivities"[tiab:~6] OR "red meat allergies"[tiab:~6] OR "red meat sensitivities"[tiab:~6] OR "red meat hypersensitivities"[tiab:~6] OR "red meat hyper sensitivities"[tiab:~6]) | 310 |
| #2 | alpha gal reaction | (("galactosyl-(1-3)galactose"[nm] OR "alpha gal"[tw] OR "a gal"[tw] OR "α gal"[tw] OR "agal"[tw] OR "αgal"[tw] OR "galili antigen*"[tw] OR "galactose-alpha-1,3-galactose"[tw] OR "galactose-α-1,3-galactose"[tw] OR "alpha galactose"[tw] OR "galactose alpha"[tw] OR "α galactose"[tw] OR "galactose α"[tw] OR "alfagal"[tw] OR "alfa gal"[tw] OR "antigal"[tw] OR "anti-gal"[tw] OR "antialphagal"[tw] OR "antiαgal"[tw]) AND ("anaphylaxis"[mesh] OR "hypersensitivity"[mesh:noexp] OR "hypersensitivity, delayed"[mesh:noexp] OR "food hypersensitivity"[mesh:noexp] OR "hypersensitivity, immediate"[mesh:noexp] OR "dermatitis, allergic contact"[mesh:noexp] OR "transfusion reaction"[mesh] OR "urticaria"[mesh:noexp] OR "allerg*"[tw] OR "antigen*"[tw] OR "anaphyla*"[tw] OR "hypersens*"[tw] OR "purpur*"[tw] OR "react*"[tw] OR "sensitiv*"[tw] OR "sensitiz*"[tw] OR "sensitis*"[tw] OR "syndrom*"[tw] OR "urticari*"[tw])) | 2,006 |
| #3 | rma OR ags | #1 OR #2 | 2,050 |
| #4 | blood | ("blood banking"[mesh] OR "blood banks"[mesh] OR "blood component transfusion"[mesh] OR "blood donors"[mesh] OR "blood group incompatibility"[mesh] OR "blood grouping and crossmatching"[mesh] OR "blood safety"[mesh] OR "blood transfusion"[mesh] OR "blood"[mesh] OR "blood"[sh] OR "hematology"[mesh] OR "plasma exchange"[mesh] OR "platelet transfusion"[mesh] OR "serum"[mesh] OR "transfusion medicine"[mesh] OR "transfusion reaction"[mesh:noexp] OR "blood*"[tw] OR "precipitate*"[tw] OR "cryoprecipitate*"[tw] OR "donat*"[tw] OR "donor*"[tw] OR "hematolog*"[tw] OR "plasma*"[tw] OR "platelet*"[tw] OR "recipient*"[tw] OR "sera"[tw] OR "sero*"[tw] OR "serum"[tw] OR "serums"[tw] OR "transfus*"[tw]) | 6,975,071 |
| #5 |  | #3 AND #4 | 1,195 |

# Embase (Embase.com)

| **Set** | **Concept** | **Search Strategy** | **Results** |
| --- | --- | --- | --- |
| #1 | red meat allergy | (('mammalian meat') NEAR/6 ('allergy' OR 'allergies' OR 'sensitivity' OR 'sensitivities' OR 'hypersensitivity' OR 'hypersensitivities')):ti,ab OR (('red meat') NEAR/6 ('allergy' OR 'allergies' OR 'sensitivity' OR 'sensitivities' OR 'hypersensitivity' OR 'hypersensitivities')):ti,ab | 333 |
| #2 | alpha gal reaction | (('galactose a 1 3 galactose':ti,ab,kw,de OR 'alpha gal':ti,ab,kw,de OR 'a gal':ti,ab,kw,de OR 'α gal':ti,ab,kw,de OR 'agal':ti,ab,kw,de OR 'αgal':ti,ab,kw,de OR 'galili antigen*':ti,ab,kw,de OR 'galactose-alpha-1 3-galactose':ti,ab,kw,de OR 'galactose-α-1 3-galactose':ti,ab,kw,de OR 'alpha galactose':ti,ab,kw,de OR 'galactose alpha':ti,ab,kw,de OR 'α galactose':ti,ab,kw,de OR 'galactose α':ti,ab,kw,de OR 'alfagal':ti,ab,kw,de OR 'alfa gal':ti,ab,kw,de OR 'antigal':ti,ab,kw,de OR 'anti-gal':ti,ab,kw,de OR 'antialphagal':ti,ab,kw,de OR 'antiαgal':ti,ab,kw,de) AND ('anaphylaxis'/exp OR 'hypersensitivity'/de OR 'delayed hypersensitivity'/de OR 'food allergy'/de OR 'immediate type hypersensitivity'/de OR 'allergic contact dermatitis'/de OR 'blood transfusion reaction'/exp OR 'urticaria'/de OR 'allerg*':ti,ab,kw,de OR 'antigen*':ti,ab,kw,de OR 'anaphyla*':ti,ab,kw,de OR 'hypersens*':ti,ab,kw,de OR 'purpur*':ti,ab,kw,de OR 'react*':ti,ab,kw,de OR 'sensitiv*':ti,ab,kw,de OR 'sensitiz*':ti,ab,kw,de OR 'sensitis*':ti,ab,kw,de OR 'syndrom*':ti,ab,kw,de OR 'urticari*':ti,ab,kw,de)) | 2,914 |
| #3 | rma OR ags | #1 OR #2 | 2,968 |
| #4 | blood | ('blood banking'/exp OR 'blood bank'/exp OR 'blood component therapy'/exp OR 'blood donor'/exp OR 'blood group incompatibility'/exp OR 'blood group typing'/exp OR 'blood safety'/exp OR 'blood transfusion'/exp OR 'blood'/exp OR 'hematology'/exp OR 'plasma exchange'/exp OR 'thrombocyte transfusion'/exp OR 'serum'/exp OR 'transfusion medicine'/exp OR 'blood transfusion reaction'/de OR 'blood*':ti,ab,kw,de OR 'precipitate*':ti,ab,kw,de OR 'cryoprecipitate*':ti,ab,kw,de OR 'donat*':ti,ab,kw,de OR 'donor*':ti,ab,kw,de OR 'hematolog*':ti,ab,kw,de OR 'plasma*':ti,ab,kw,de OR 'platelet*':ti,ab,kw,de OR 'recipient*':ti,ab,kw,de OR 'sera':ti,ab,kw,de OR 'sero*':ti,ab,kw,de OR 'serum':ti,ab,kw,de OR 'serums':ti,ab,kw,de OR 'transfus*':ti,ab,kw,de) | 10,471,120 |
| #5 |  | #3 AND #4 | 1,946 |

# Cochrane CDSR and CENTRAL (Wiley)

| **Set** | **Concept** | **Search Strategy** | **Results** |
| --- | --- | --- | --- |
| #1 | red meat allergy | (("mammalian meat":ti,ab) NEAR/6 ("allergy":ti,ab OR "allergies":ti,ab OR "sensitivity":ti,ab OR "sensitivities":ti,ab OR "hypersensitivity":ti,ab OR "hypersensitivities":ti,ab)) OR (("red meat":ti,ab) NEAR/6 ("allergy":ti,ab OR "allergies":ti,ab OR "sensitivity"ti,ab OR "sensitivities":ti,ab OR "hypersensitivity":ti,ab OR "hypersensitivities":ti,ab)) | 6 |
| #2 | alpha gal reaction | ((("galactosyl":ti,ab NEXT "galactose":ti,ab) OR "alpha gal":ti,ab OR "a gal":ti,ab OR "α gal":ti,ab OR "agal":ti,ab OR αgal:ti,ab OR ("galili":ti,ab NEXT antigen*:ti,ab) OR ("galactose":ti,ab NEXT "galactose":ti,ab) OR "alpha galactose":ti,ab OR "galactose alpha":ti,ab OR "α galactose":ti,ab OR "galactose α":ti,ab OR "alfagal":ti,ab OR "alfa gal":ti,ab OR "antigal":ti,ab OR "anti-gal":ti,ab OR "antialphagal":ti,ab OR "antiαgal":ti,ab) AND ([mh "anaphylaxis"] OR [mh ^"hypersensitivity"] OR [mh ^"hypersensitivity, delayed"] OR [mh ^"food hypersensitivity"] OR [mh ^"hypersensitivity, immediate"] OR [mh ^"dermatitis, allergic contact"] OR [mh "transfusion reaction"] OR [mh ^"urticaria"] OR allerg*:ti,ab OR antigen*:ti,ab OR anaphyla*:ti,ab OR hypersens*:ti,ab OR purpur*:ti,ab OR react*:ti,ab OR sensitiv*:ti,ab OR sensitiz*:ti,ab OR sensitis*:ti,ab OR syndrom*:ti,ab OR urticari*:ti,ab)) | 40 |
| #3 | rma OR ags | #1 OR #2 | 46 |
| #4 | blood | ([mh "blood banking"] OR [mh "blood banks"] OR [mh "blood component transfusion"] OR [mh "blood donors"] OR [mh "blood group incompatibility"] OR [mh "blood grouping and crossmatching"] OR [mh "blood safety"] OR [mh "blood transfusion"] OR [mh blood] OR [mh hematology] OR [mh "plasma exchange"] OR [mh "platelet transfusion"] OR [mh serum] OR [mh "transfusion medicine"] OR [mh ^"transfusion reaction"] OR blood*:ti,ab,kw OR precipitate*:ti,ab,kw OR cryoprecipitate*:ti,ab,kw OR donat*:ti,ab,kw OR donor*:ti,ab,kw OR hematolog*:ti,ab,kw OR plasma*:ti,ab,kw OR platelet*:ti,ab,kw OR recipient*:ti,ab,kw OR sera:ti,ab,kw OR sero*:ti,ab,kw OR serum:ti,ab,kw OR serums:ti,ab,kw OR transfus*:ti,ab,kw) | 618,493 |
| #5 |  | #3 AND #4 | 29 |
|  |  | CENTRAL: 29  CDSR: 0 |  |

# Web of Science Core Collection

- WoS Core Collection Includes: SCI-EXPANDED (1900 – present), SSCI (1900 – present), CPCI-S (1990 – present), CPCI-SSH (1990 – present), BKCI-S (2005 – present), BKCI-SSH (2005 – present), ESCI (2005 – present), CCR-EXPANDED (1985 – present), IC (1993 – present)

| **Set** | **Concept** | **Search Strategy** | **Results** |
| --- | --- | --- | --- |
| #1 | red meat allergy | TS=(("mammalian meat") NEAR/6 ("allergy" OR "allergies" OR "sensitivity" OR "sensitivities" OR "hypersensitivity" OR "hypersensitivities")) OR TS=(("red meat") NEAR/6 ("allergy" OR "allergies" OR "sensitivity" OR "sensitivities" OR "hypersensitivity" OR "hypersensitivities")) | 342 |
| #2 | alpha gal reaction | (TS=(("galactosyl" NEAR "galactose") OR ("galactose" NEAR "galactose") OR "alpha gal" OR "a gal" OR "α gal" OR "agal" OR "αgal" OR "galili antigen*" OR "galactose-alpha-1,3-galactose" OR "galactose-α-1,3-galactose" OR "alpha galactose" OR "galactose alpha" OR "α galactose" OR "galactose α" OR "alfagal" OR "alfa gal" OR "antigal" OR "anti-gal" OR "antialphagal" OR "antiαgal") AND TS=("anaphylaxis" OR "hypersensitivity" OR "transfusion reaction" OR "urticaria" OR "allerg*" OR "antigen*" OR "anaphyla*" OR "hypersens*" OR "purpur*" OR "react*" OR "sensitiv*" OR "sensitiz*" OR "sensitis*" OR "syndrom*" OR "urticari*")) | 14,960 |
| #3 | rma" OR "ags | #1 OR #2 | 15,036 |
| #4 | blood | TS=("blood banking" OR "blood banks" OR "blood component transfusion" OR "blood donors" OR "blood group incompatibility" OR "blood" OR "blood safety" OR "blood transfusion" OR "blood" OR "hematology" OR "plasma exchange" OR "platelet transfusion" OR "serum" OR "transfusion medicine" OR "transfusion reaction" OR "blood*" OR "precipitate*" OR "cryoprecipitate*" OR "donat*" OR "donor*" OR "hematolog*" OR "plasma*" OR "platelet*" OR "recipient*" OR "sera" OR "sero*" OR "serum" OR "serums" OR "transfus*") | 6,967,723 |
| #5 |  | #3 AND #4 | 4,586 |

# Scopus (Elsevier)

| **Set** | **Concept** | **Search Strategy** | **Results** |
| --- | --- | --- | --- |
| #1 | red meat allergy | TITLE-ABS(("mammalian meat") W/6 ("allergy" OR "allergies" OR "sensitivity" OR "sensitivities" OR "hypersensitivity" OR "hypersensitivities")) OR TITLE-ABS(("red meat") W/6 ("allergy" OR "allergies" OR "sensitivity" OR "sensitivities" OR "hypersensitivity" OR "hypersensitivities")) | 210 |
| #2 | alpha gal reaction | (TITLE-ABS(("galactosyl" W/3 "galactose") OR ("galactose" W/3 "galactose") OR "alpha gal" OR "a gal" OR {α gal} OR "agal" OR {αgal} OR "galili antigen*" OR "alpha galactose" OR "galactose alpha" OR {α galactose} OR "galactose " OR "alfagal" OR "alfa gal" OR "antigal" OR "anti-gal" OR "antialphagal" OR {antiαgal}) AND TITLE-ABS("anaphylaxis" OR "hypersensitivity" OR "transfusion reaction" OR "urticaria" OR "allerg*" OR "antigen*" OR "anaphyla*" OR "hypersens*" OR "purpur*" OR "react*" OR "sensitiv*" OR "sensitiz*" OR "sensitis*" OR "syndrom*" OR "urticari*")) | 13,580 |
| #3 | rma OR ags | #1 OR #2 | 13,635 |
| #4 | blood | TITLE-ABS("blood banking" OR "blood banks" OR "blood component transfusion" OR "blood donors" OR "blood group incompatibility" OR "blood grouping and crossmatching" OR "blood safety" OR "blood transfusion" OR "blood" OR "hematology" OR "plasma exchange" OR "platelet transfusion" OR "serum" OR "transfusion medicine" OR "transfusion reaction" OR "blood*" OR "precipitate*" OR "cryoprecipitate*" OR "donat*" OR "donor*" OR "hematolog*" OR "plasma*" OR "platelet*" OR "recipient*" OR "sera" OR "sero*" OR "serum" OR "serums" OR "transfus*") | 7,117,552 |
| #5 |  | #3 AND #4 | 3,976 |
