# Supplementary material for: Alpha-gal sensitization and allergic blood transfusion reactions: a scoping review
Source: J Transl Med. 2026 Feb 4;24:325. doi: 10.1186/s12967-025-07614-9 (PMC12964899; doi:10.1186/s12967-025-07614-9)
Supplement: Supplementary file 3 — Supplementary Material 3 [file 12967_2025_7614_MOESM3_ESM.docx]

1. Skip to main content

# Data Extraction Template

Published You published 4 months ago.


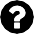


Save as Draft

Publish

# Item Settings Editor

Reset template and delete all data


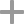

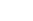


General information


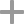

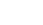


Study ID


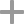

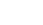


Title


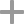

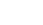


Publisher (Name of Journal, University, etc.)


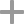

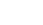


Corresponding author contact details


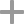

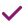


Geographic areas in which the study was conducted

1. –

North America

1. –

Central and South America

1. –

Europe

1. –

Asia

1. –

Africa

1. –

Australia and New Zealand

1. –

Other


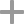

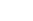


Study characteristics


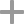

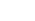


Methods


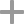

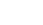


Aim(s)/objectives of study


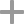

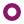


Study design

1. –

Randomised controlled trial

2. –

Non-randomised experimental study

3. –

Cohort study

4. –

Cross sectional study

5. –

Case control study

6. –

Systematic review

7. –

Qualitative research

1. –

Prevalence study

1. –

Case series

1. –

Case report

1. –

Diagnostic test accuracy study

1. –

Clinical prediction rule

1. –

Economic evaluation

1. –

Text and opinion

1. –

Other


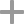

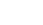


Start date (of data collection)


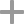

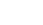


End date (of data collection)


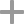

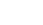


Study funding sources


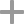

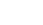


Possible conflicts of interest for study authors


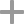

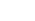


Participants


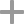

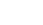


Population description


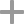

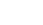


Inclusion criteria


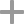

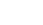


Exclusion criteria


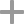

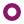


Method of recruitment of participants

1. –

Phone

2. –

Mail

3. –

Clinic patients

4. –

Voluntary

5. –

Other


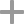

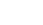


Total number of participants


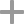

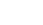


Baseline Population Characteristics


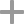

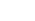


Results


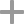

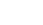


Major Results


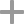

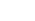


Conclusions and Impressions


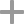

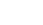


Conclusion


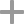

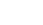


Study Strengths


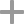

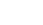


Study Weaknesses


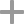

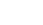


Miscellaneous Notes

# Preview

**General information**

Study ID

If Covidence autopopulates an ID, use that. If not, leave blank and ask Dr. Miller for instructions.

Title

Title of paper / abstract / report that data are extracted from

Publisher (Name of Journal, University, etc.)

Corresponding author contact details Email and phone from paper OK

Geographic areas in which the study was conducted

Please check boxes for geographic regions from which the data were collected. List individual country/countries in Notes if not covered in list.

1.
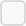
 North America
2.
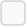
 Central and South America
3.
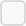
 Europe
4.
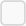
 Asia
5.
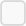
 Africa
6.
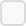
 Australia and New Zealand
7.
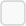
 Other

# Study characteristics Methods

Aim(s)/objectives of study

OK to copy aim(s) directly from the paper.

Study design

Check most applicable option and enter "other" if study is mixed methods or includes multiple types of studies.

1.
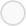
 Randomised controlled trial
2.
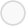
 Non-randomised experimental study
3.
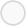
 Cohort study
4.
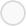
 Cross sectional study
5.
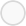
 Case control study
6.
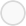
 Systematic review
7.
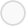
 Qualitative research
8.
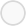
 Prevalence study
9.
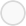
 Case series
10.
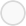
 Case report
11.
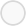
 Diagnostic test accuracy study
12.
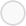
 Clinical prediction rule
13.
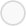
 Economic evaluation
14.
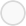
 Text and opinion
15.
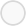
 Other

Start date (of data collection)

End date (of data collection)

Study funding sources

Possible conflicts of interest for study authors

# Participants

Population description

Inclusion criteria

Exclusion criteria

Method of recruitment of participants

Choose most frequently used option. If recruitment requires more explanation, put in "Other."

1.
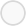
 Phone
2.
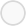
 Mail
3.
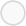
 Clinic patients
4.
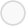
 Voluntary
5.
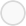
 Other

Total number of participants

Baseline Population Characteristics

Copy from methods or results section (e.g., cohort demographics).

# Results

Major Results

Record major results here (usually found in abstract, results, or first paragraph of discussion). If the associated tables are too complex to reproduce, write "See Table X in paper" or whatever else is most appropriate.

Unfortunately, this form cannot do images or screenshots of the tables.

# Conclusions and Impressions

Conclusion

Copy the conclusion from the abstract or the discussion.

Study Strengths

OK to use the authors' impressions or your own. If you quote the authors, please put the information in quotation marks.

Study Weaknesses

OK to use the authors' impressions or your own. If you quote the authors, please put the information in quotation marks.

Miscellaneous Notes

Add any relevant information not covered in the fields above. OK to leave blank if you have no additional comment.

**Feedback & Support**

**Find your answer, fast.**

We've got a new Knowledge base that answers many frequently asked questions. Why not try that first? [View the knowledge base](http://support.covidence.org/help_center) OR Send us an email
